# Dasatinib ameliorates thioacetamide-induced liver fibrosis: modulation of miR-378 and miR-17 and their linked Wnt/β-catenin and TGF-β/smads pathways

**DOI:** 10.1080/14756366.2021.1995379

**Published:** 2021-12-11

**Authors:** Mai A. Zaafan, Amr M. Abdelhamid

**Affiliations:** a Faculty of Pharmacy, Pharmacology and Toxicology Department, October University for Modern Sciences and Arts (MSA), Dokki, Egypt; b Faculty of Pharmacy, Biochemistry Department, October University for Modern Sciences and Arts (MSA), Dokki, Egypt

**Keywords:** Dasatinib, liver fibrosis, smad-3, Wnt-10, mice

## Abstract

Hepatic stellate cells activation (HSCs) plays a crucial role in the pathogenesis of liver fibrosis. Specific microRNAs have been suggested to affect the activation of HSCs via various signalling pathways including TGF-β/smads and Wnt/β-catenin pathways. Dasatinib is a multitarget inhibitor of many tyrosine kinases has recently studied for its anti-fibrotic effects in a variety of fibrous diseases. This study investigated the role of modulation of miRNA-378 and miRNA-17 in the pathogenesis of liver fibrosis through altering Wnt/β-catenin and TGF-β/smads pathways and evaluated the beneficial effect of the tyrosine kinase inhibitor, dasatinib, in thioacetamide-induced liver fibrosis model in mice. Treatment with dasatinib down-regulated miRNA-17 expression, leading to the restoration of WiF-1 and smad-7 which cause the inhibition of both Wnt/β-catenin and TGF-β/smads signalling. In addition, it upregulated miRNA-378 leading to the decrease of Wnt-10 which contributes to the suppression of activated HSCs.

## Graphical Abstract

**Figure:**
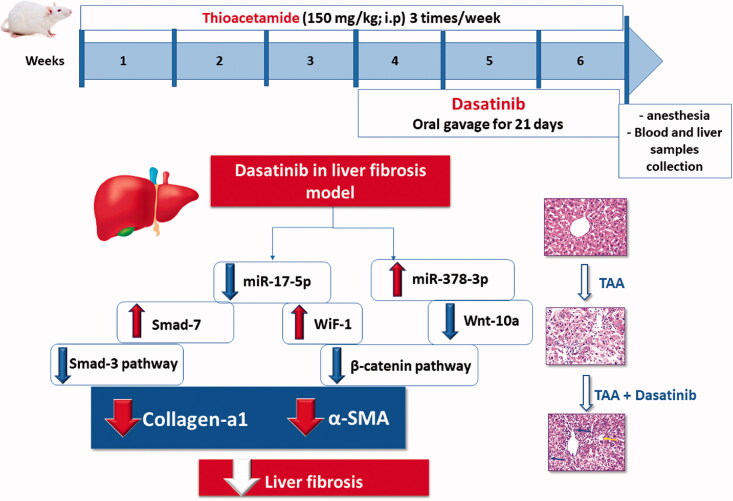

## 1. Introduction

Chronic liver diseases (CLDs) are a major public health issue that affects people all over the world. CLDs affect 844 million people worldwide, resulting in two million deaths each year, according to estimates^1^. Liver fibrosis is characterised by an excessive deposition of extracellular matrix (ECM) proteins in liver, mainly synthesised by activated hepatic stellate cells (HSCs)^2^. Hundreds of thousands of people worldwide suffer from liver fibrosis, which is caused in part by the obesity epidemic, as well as the high prevalence of alcohol addiction and viral hepatitis^3^. Hepatitis C virus (HCV) is the leading cause of chronic hepatitis, liver cirrhosis and hepatocellular carcinoma (HCC). Around 55–85% of HCV-infected patients become chronic active cases and go through the stages of fibrosis, cirrhosis, and possibly HCC^4^.

TGF-β/smad, p38 MAPK, and other pathways have all been linked to the development of liver fibrosis. However, the molecular mechanism is unknown, and there is no effective treatment available^5^. Exploration of new signal pathways and the development of novel therapeutic strategies are thus urgently required. Previous studies have shown that an abnormal Wnt/β-catenin signalling pathway plays a key role in the development of organ fibrosis, accelerates HSC activation including cell proliferation and ECM accumulation, and may be a novel therapeutic target in fibrotic disorders^6^. Single Wnt ligands can activate multiple signalling pathways, increased gene expression of Wnt-1, Wnt-10, and β-catenin were observed in lung fibrosis^7^. Overexpression of Wnt-10 results in progressive loss of subcutaneous adipose tissue accompanied by dermal fibrosis and increased expression of fibrotic genes^8^. Several secreted protein families antagonise Wnt/β-catenin signalling. The function of Wnt inhibitors depends on their expression levels and the cellular context. The secreted Frizzled-related proteins (sFRPs) and Wnt inhibitory factor (WIF), which exhibit a high degree of homology with the Wnt ligand-binding domains of Fzd, both bind to Wnt ligands, and thereby function as Wnt antagonists for both β-catenin and noncanonical signalling^9^.

MicroRNAs are short RNA sequences that regulate gene expression by destabilising mRNA and inhibiting mRNA translation^10^. Specific microRNAs have been recognised to play a role in the activation of HSCs through a variety of signalling pathways^11^^,^^12^. HSC activation involves many signalling pathways such as TGFβ/smads signalling and Wnt/β-catenin pathway^13^. MiR-17 is reported to synergistically trigger fibrosis development via its target genes smad-7^14^ and WIFI^15^. MiR-17 is increased in carbon tetrachloride (CCl4)-treated rat liver fibrotic tissues due to the negative role of smad-7 in TGF-β/smad signalling. Inhibition of miR-17 suppressed proliferation of HSCs, ECM production and α-smooth muscle actin (α-SMA) expression induced by TGF-β1^16^. In addition, overexpression of miR-17–5 and suppression of WIFI enhanced the Wnt/β-catenin pathway in liver fibrotic tissues; earlier research has shown that WIFI is a direct downstream target of miR-17^15^^,^^17^. Recently, miR-378a has been reported to be down-regulated in fibrotic liver tissues and inhibits HSC activation via targeting of Wnt-10^18^. Overexpression of miR-378a resulted in the suppression of HSC activation including HSC proliferation, α-SMA and type-I collagen^19^.

Dasatinib is a second-generation oral multitarget inhibitor of many tyrosine kinases^10^^,^^20^. Dasatinib was designed to treat some types of cancers including chronic myeloid leukaemia (CML)^21^. Dasatinib has recently been studied for its anti-fibrotic effects in a variety of fibrous diseases, including systemic sclerosis, lung fibrosis, and chronic pancreatitis. Through the TKs/GSK3/β-catenin pathway, dasatinib inhibits the proliferation and activation of pancreatic stellate cells (PSCs)^21^^,^^22^. The current study aims to investigate the potential efficacy and the molecular mechanisms of dasatinib in the treatment of thioacetamide-induced liver fibrosis by modulating miR-378 and miR-17 via the Wnt/β catenin and TGF-/smad pathways.

## 2. Materials and methods

### 2.1. Animals

Male albino mice weighing between 15 and 20 g were used in this study. The Egyptian Company for the Production of Vaccines, Sera, and Drugs provided these mice (EGYVAC; Cairo, Egypt). Mice were housed in plastic cages at October University for Modern Sciences and Arts’ animal house under constant conditions (temperature 25 ± 3 °C and humidity 50%). Free water and standard pellet chow (El-Nasr Co., Egypt) were available. The study was approved from the ethics committee of October University for Modern Sciences and Arts.

### 2.2. Drugs and chemicals

Dasatinib and thioacetamide were purchased from Sigma-aldrich (Saint Louis, MO, USA). The other chemicals used were all of analytical grade.

### 2.3. Induction of liver fibrosis

For the induction of liver fibrosis, thioacetamide (150 mg/kg; i.p) dissolved in saline was injected three times a week for 6 weeks. The method of induction of liver fibrosis was chosen based on previous research^23^^,^^24^.

### 2.4. Experimental design

Mice were categorised into three groups (*n* = 6) at random. The first set of mice served as the normal control group. thioacetamide (150 mg/kg; i.p.) was given to the liver fibrosis control group. The third group was treated with dasatinib (20 mg/kg/day; p.o.) for 21 days starting from the 4th week of the experiment. Based on previous research, the dose and route of administration of dasatinib were determined^22^.

At the end of the 6th week, blood samples were collected via the retro-orbital plexus for serum separation and liver enzymes investigation. Liver enzymes were analysed using commercial kits (Biodiagnostic; cairo, Egypt).

The mice were then sacrificed via cervical dislocation under ether anaesthesia, and the livers were quickly dissected out and washed in ice-cold saline. RNA extraction from the isolated livers were used for analysis of the expression of miR-378-3p, miR-17-5p, Wnt-10a, WiF-1, β-catenin, smad-7, smad-3 and collagen-a1 through qRT-PCR. Sections of the isolated livers were fixed in formalin and used for the histopathological examination as well as the investigation of the immunohistochemical reactivity of TNF-α and α-SMA.

### 2.5. Quantitative real-time polymerase chain reaction (RT-PCR)

The isolated livers were used for total RNA isolation using Trizol (Invitrogen; Auckland, *New Zealand*), according to the manufacturer’s instructions and reverse-transcribed into cDNA with the Reverse Transcriptase M-MLV (Promega, Madison, WI, USA).

Primer sequences to be used in the experiment were as follows:

For miRNA quantitative reverse transcriptase PCR, small RNA species-enriched RNA was isolated according to the manufacturer’s instructions (mirVana miRNA isolation kit; Ambion, Austin, TX, USA). miRNA was reverse-transcribed by using Ncode miRNA first-strand complementary DNA synthesis kits (Invitrogen). Quantitative reverse transcriptase PCR was performed by using a Power SYBR Green PCR Master Mix on the CFX96 Instrument (Bio-Rad, USA). Data analysis was determined by using the relative standard curve method.

**Table:** 

Genea	Forward primer	Reverse primer
U6	CGCTTCGGCAGCACATATACTA	CGCTTCACGAATTTGCGTGTCA
Wnt-10a	GATGGTGGGGCATCGTGAA	GGGTTCTGTCGGATCAGTCG
Col1a1	TAACTTCTGGAATTCGACTTT TTGG	GTCCAGCCCTCATCCTGGCC
Smad-7	CTCGGACAGCTCAATTCGGA	CAGTGTGGCGGACTTGATGA
Smad-3	TGGCCACTGCTGCTTCCTCTTCTT	GGGGCC AGCTTCGTCATACTCCT
WIF-1	GAATTTTACCTGGCAAGCTGCGG	GACGGGCTTAGAGCACAGGTCTCC
β-catenin	CCGTTCGCCTTCATTATGGA	GGCAAGGTTTCGAATCAATCC
miR 17-5p	TCTAGATCCCGAGGACTG	ATCGTGACCTGAA
miR-378a-3p	TGGGGACTGGACTTGGAGTC	GTCGTATCCAGTGCAGGGTCCG AGGTATTCGCACTGGATACGAC CCTTCT
β-actin	CTGAGAGGGAAATCGTGCGT	TTGTTGGCATAGAGGTCTTTA

### 2.6. Histopathologic assessment of hepatic tissue damage

The livers from the different groups were fixed in 10% formalin solution. Sections of the livers were collected on glass slides, deparaffinised and stained by haematoxylin and eosin stain for routine histopathological examination using electric light microscope. This is according to the method previously described by JD Bancroft and M Gamble^25^.

### 2.7. Immunohistochemical reaction of TNF-α and α-SMA

Sections from liver tissue of around 3 µm thickness embedded in paraffin were used for detection of TNF-α and α-SMA through the immunostaining with primary antibody polyclonal immunoglobulin-G of mice TNF-α and α-SMA according the method previously described by^26^. Finally, grading of immunohistochemical reactivity was measured from four randomly chosen fields in each section and averaged using image analysis software (Image J, Fiji version; MD, USA).

### 2.8. Statistical analysis

Data are presented in the form of mean ± SEM. The comparisons among means of different groups were done via one-way analysis of variance (ANOVA) and Tukey–Kramer multiple comparisons post-test ^27^. Kruskal–Wallis test was used for analysing the histopathological scores and followed by Dunn’s multiple comparisons test. The level of significance was taken as *p* ˂ .05. All the statistical tests carried out using GraphPad Prism software package, version 5 (GraphPad Software, Inc., USA).

## 3. Results

### 3.1. Effect of dasatinib on serum levels of liver enzymes in liver fibrosis

**Figure 1.**
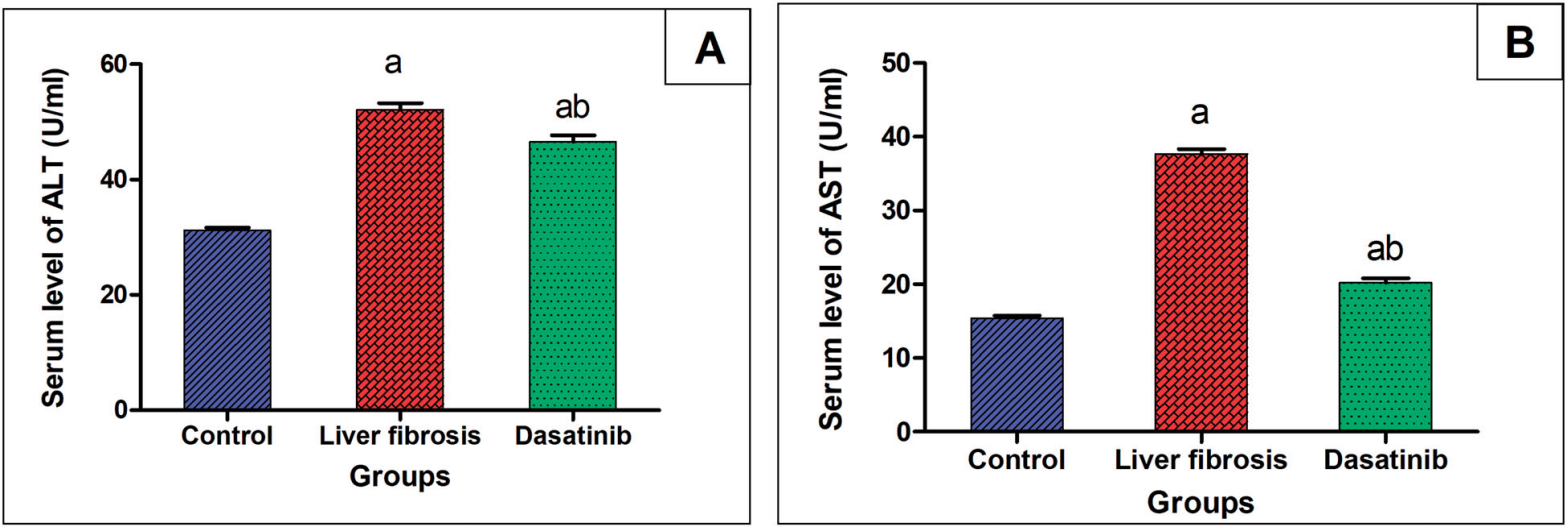
(A, B) Effect of dasatinib treatment on serum levels of the liver enzymes; alanine transaminase (ALT) and aspartate transaminase (AST) in mice with thioacetamide-induced liver fibrosis. The data are presented as mean ± SEM (*n* = 6). ^a^Significant difference from the control group; ^b^significant difference from liver fibrosis-inducted group (at *p* ˂ .05)

Thioacetamide resulted in significant increase in the serum levels of liver enzymes. Alanine transaminase (ALT) was increased by 1.67-fold and aspartate transaminase was increased by 2.44-fold in the inducted liver fibrosis group compared to the control group. On the other hand, treatment with dasatinib significantly lowered the serum levels of ALT by 10.67% and AST by 46.32% compared to the mice with liver fibrosis (Figure 1).

### 3.2. Effect of dasatinib on miR-378 and Wnt-10a/β-catenin signalling in liver fibrosis

**Figure 2.**
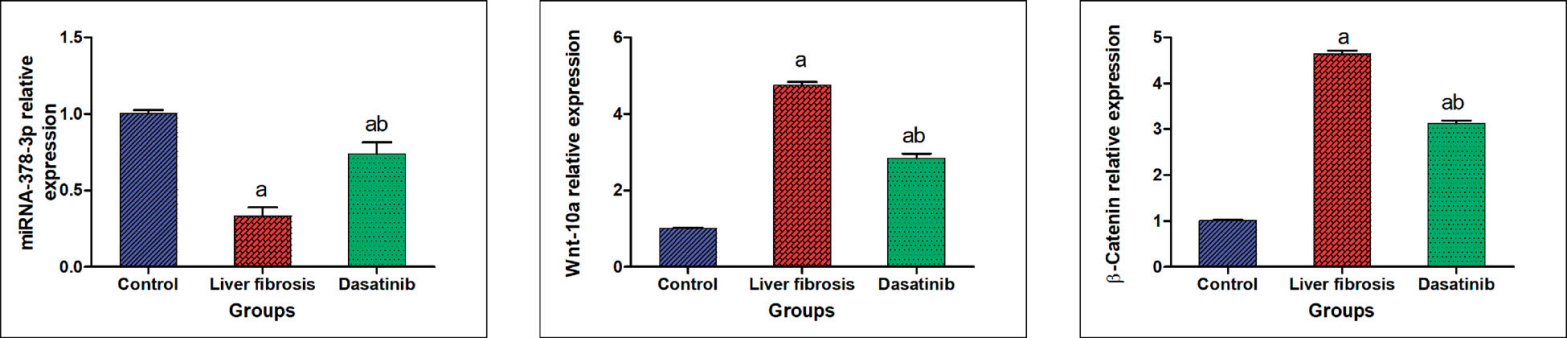
Effect of dasatinib treatment on relative expression of miRNA-378-3p, Wnt-10a and β-catenin in the liver tissue of mice with thioacetamide-induced liver fibrosis. The data are presented as mean ± SEM (*n* = 6). ^a^Significant difference from the control group; ^b^significant difference from liver fibrosis-inducted group (at *p* ˂ .05).

The mice with liver fibrosis exhibited a significantly suppressed expression of miR-378-3p in the liver (with a 67% decrease compared to the control group). This effect was accompanied by a significantly elevated expression of Wnt-10a and β-cantenin (475 and 4.64-fold elevation compared to the control group). These effects were abolished by treatment with dasatinib which significantly increased the liver expression level of miR-378-3p with 2.24-fold and decreased the expression level of Wnt-10a and β-cantenin in the liver by 40.0% and 32.5%, respectively compared to the mice with liver fibrosis (Figure 2).

### 3.3. Effect of dasatinib on miR-17 and smad-7/smad-3 signalling in liver fibrosis

**Figure 3.**
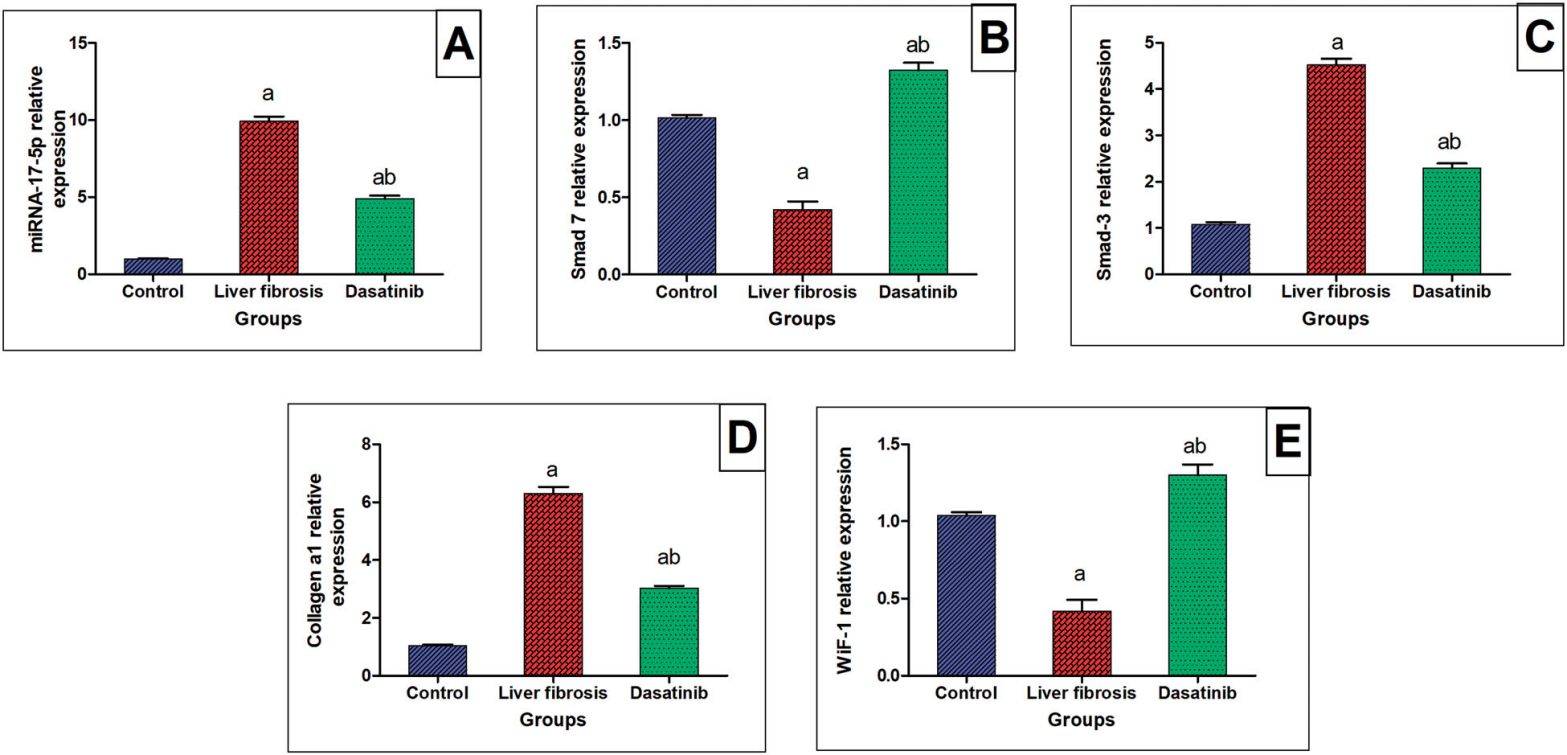
(A–E) Effect of dasatinib treatment on relative expression of miRNA-17-5p, smad-7, smad-3, collagen a1 and Wnt inhibitory factor-1 (WIF-1) in liver tissue of mice with thioacetamide-induced liver fibrosis. The data are presented as mean ± SEM (*n* = 6). ^a^Significant difference from the control group; ^b^significant difference from liver fibrosis inducted-group (at *p* ˂ .05).

A significant 9.9-fold elevation was observed in the hepatic level of miR-17-5p in the liver fibrosis group compared to the control group. On the other hand, treatment with dasatinib significantly lowered the hepatic level of miR-17-5p by 50.5% compared with the liver fibrosis mice (Figure 3(A)).

In addition, the results of the current study indicated that the hepatic level of smad-7 was significantly reduced by 58.0%, whereas hepatic levels of smad-3 and collagen a1 were raised by 4.5- and 6.3-fold, respectively in the liver fibrosis group compared to the control group. Conversely, treatment with dasatinib resulted in a significant 3.1-fold elevation in the hepatic level of smad-7 and a significant decline in smad-3 and collagen a1 by 49.2% and 51.9%, respectively compared to the liver fibrosis group (Figure 3(B–D)).

Furthermore, the impact of the modulation of miR-17 was reflected on the hepatic expression level of WiF-1 that is significantly reduced by 62% in the liver fibrosis group compared to the control group. Treatment with dasatinib significantly elevated the hepatic expression level of Wif-1 by 3.09-fold compared to the liver fibrosis group (Figure 3(E)).

### 3.4. Effect of dasatinib on immunohistochemical reactivity of TNF-α and α-SMA in liver fibrosis

**Figure 4.**
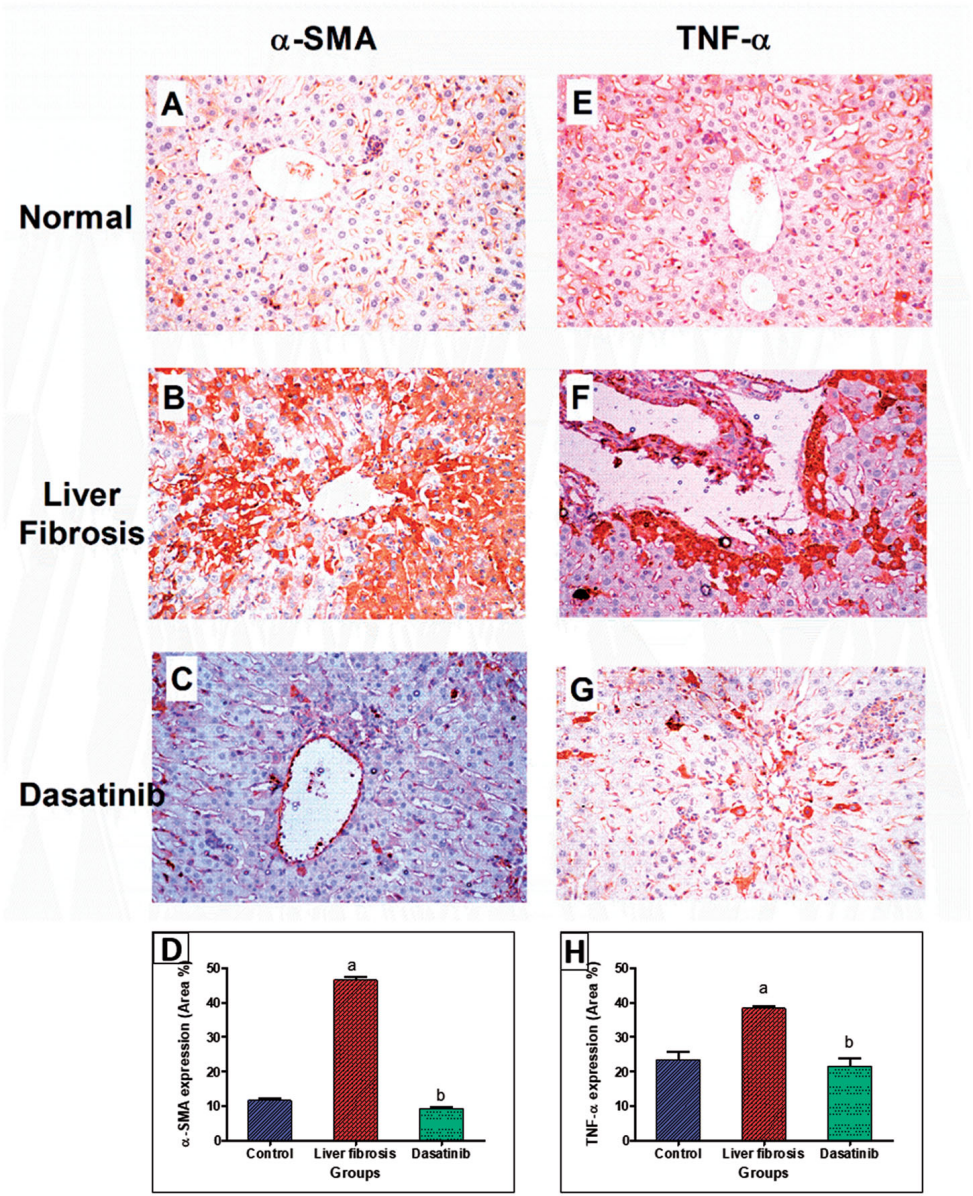
Immunostaining of α-smooth muscle actin (α-SMA) and tumour necrosis factor-α (TNF-α) in the liver tissue of mice with thioacetamide-induced liver fibrosis (H&E × 40). (A) α-SMA/control group, (B) α-SMA/liver fibrosis group, (C) α-SMA/dasatinib-treated group, (E) TNF-α/control group, (F) TNF-α/liver fibrosis group, (G) TNF-α/dasatinib-treated group, (D, H) represent a comparative quantification of the immunohistochemical expression for α-SMA and TNF-α in hepatic tissue of mice from all groups: The severity of the immunoactivity is depending on the intensity and distribution of the brown colour. ^a^Represents a significant difference from the normal control group, ^b^a significant difference from liver fibrosis inducted-group (at *p* ˂ .05).

Liver sections from normal control mice (Figure 4(A)) showed relatively negative expression of α-SMA. Thioacetamide resulted in significantly increased expression of α-SMA in the hepatic parenchyma (Figure 4(B)). The group treated with dasatinib showed only weak expression of α-SMA in the hepatic tissue (Figure 4(C)) with no significant difference from the control group. The immuno-staining for TNF-α revealed weak expression in the hepatic tissue of the normal control mice (Figure 4(E)). The expression of TNF-α increased markedly in the hepatocytes surrounding the central vein and the hepatocytes surrounding the portal area upon induction of liver fibrosis (Figure 4(F)). Hepatic tissue from the dasatinib-treated mice showed noticeable suppression in TNF-α expression (Figure 4(G)). Comparative quantification of the immunohistochemical expression for α-SMA and TNF-α in hepatic tissue of mice from all groups is presented in Figure 4(D,H) expressed as area % of the brown colour according to image J software.

### 3.5. Effect of dasatinib treatment on histopathological alterations of liver

**Figure 5.**
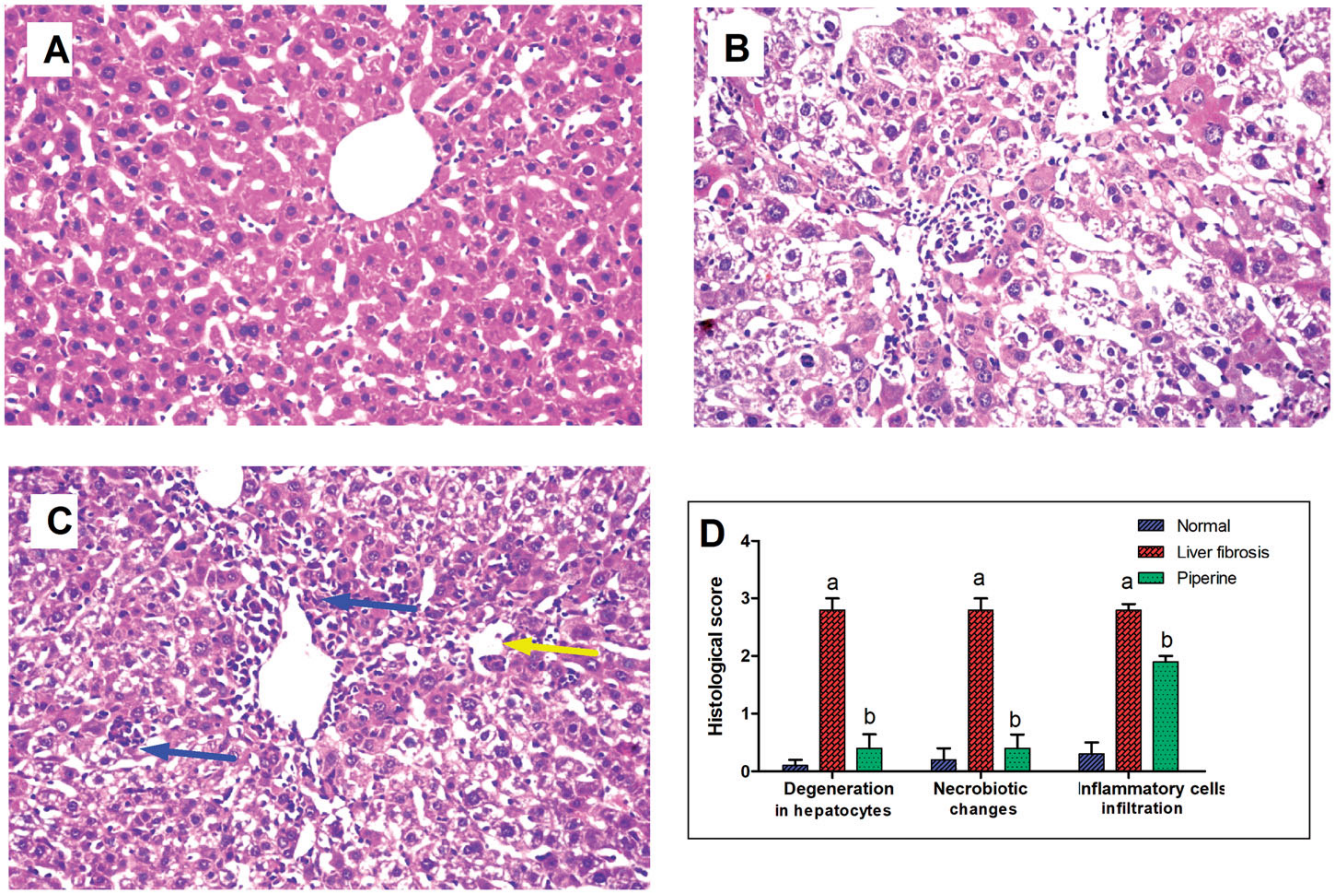
Effect of dasatinib treatment on the histopathological alterations in the liver tissue in mice with thioacetamide-induced liver fibrosis (H&E × 16): (A) control group, (B) liver fibrosis group, (C) dasatinib-treated group, (D) scoring of the histological observations in the hepatic tissue from all groups. Data are presented as mean ± SEM of 6 random non-overlapping fields/section. ^a^Significant difference from the control group, ^b^significant difference from liver fibrosis inducted-group (at *p* ˂ .05).

Histological examination of liver sections from control mice revealed normal histological structures of the central vein and the surrounding hepatocytes on the parenchyma with no histopathological alteration (Figure 5(A)). Liver sections from mice received thioacetamide alone showed degeneration and necrobiotic changes observed in the hepatocytes and associated with focal inflammatory cells infiltration in a diffuse manner in between (Figure 5(B)). Treatment with dasatinib resulted in the presence of minor degeneration in some hepatocytes with marked improvement from the liver fibrosis-inducted group in addition to the presence of some inflammatory cells infiltration (Figure 5(C)). Scoring of the histological observations in the hepatic tissue is presented in Figure 5(D).

## Discussion

Aside from the TGF/smads signalling pathway^28^, the Wnt/β-catenin pathway has been shown to play a role in stellate cell/fibroblast activation and fibrosis in a number of organs, including the liver, kidney, lung, and pancreas, making it a possible therapeutic target for liver fibrosis. Some microRNAs are supposed to be essential regulators of these signalling pathways and these hepatic stellate cells activation (HSCs)^28–30^. The present study hypothesised that dasatinib would attenuate liver fibrosis and ameliorate the inflammatory responses. For this purpose, we investigated the potential efficacy and mechanisms of dasatinib in the treatment of thioacetamide-induced liver fibrosis in mice through the modulation of miR-378 and miR-17 that can target Wnt-10 and WIF1, respectively, and inhibit the Wnt/β-catenin pathway.

Many biological processes are controlled by the interaction of receptor tyrosine kinase signalling with Wnt/β-catenin signalling, but the mechanisms of this interaction are still unknown. The role of various receptor tyrosine kinase systems in activating canonical Wnt signalling is suggested by the potent activation of Wnt/β-catenin by FGFR2, FGFR3, EGFR, and TRKA kinases.^31^ Dasatinib is a second-generation oral multitarget inhibitor of multiple tyrosine kinases that was originally designed to treat CML. Dasatinib was recently investigated for having an anti-fibrotic effect in some of the fibrous diseases, including systemic sclerosis, lung, and pancreatic fibrosis^22^^,^^32^. Dasatinib inhibited TGF-induced myofibroblast differentiation and ECM fibronectin expression in both HFLFs and NIH3T3 cells, according to Abdalla et al^33^.

Generally, HSC activation is characterised by the accumulation of collagens, the enhancement of α-SMA expression and the increase of cell proliferation^34^. Wnt-10 overexpression results in progressive loss of subcutaneous adipose tissue, dermal fibrosis, and up-regulation of fibrotic gene expression, which increased collagen aggregation and α-SMA expression^22^. MicroRNAs, have been implicated in the pathogenesis of many diseases^35^. In the current study, we observed that miR-378a expression was markedly decreased in fibrotic liver tissues, while Wnt-10 expression increased significantly. Furthermore, the dasatinib-treated group revealed a significant increase in the miR-378 expression accompanied by marked suppression of Wnt-10 expression in liver tissue. Thus, we suggest that dasatinib can suppress the HSCs activation which is a critical event in the development of liver fibrosis through the markedly increased expression of miR-378a accompanied by the suppression of Wnt-10 expression. Our finding is matched with another study reported that miR-378a was down-regulated accompanied by increased activation of HSCs in rats with CCl4-induced liver fibrosis^18^. In the current study, the increased expression of miR-378a-3p upon treatment with dasatinib can have a direct role in suppression of the activated HSCs. This effect was reflected through the significantly decreased levels α-SMA and type-I collagen as markers for HSCs activation.

Aberrant Wnt/β-catenin pathway contributes to the development of liver fibrosis^36^. Wnt/β-catenin pathway activation contributes also to HSC activation and ECM accumulation^37^. In this study, we demonstrated that miR-17 expression was increased in fibrotic liver tissues, along with a marked reduction in WIF1 and smad-7 expression levels. Dasatinib inhibits miR-17, resulting in increased expression of both WIF1 and smad-7, which are miR-17’s targets. Our findings are consistent with previous research that found WIF1, a Wnt antagonist, can reduce hepatic fibrosis by inhibiting the Wnt/β-catenin pathway^38^. WIF1 was predicted to be a putative target of miR-17, which induced HSC activation, according to Peng et al^39^. Our findings are supported by Yu et al. study, which found that miR-17 promotes HSC activation by reducing smad-7, implying that it may be useful as a new therapeutic target for liver fibrosis^14^. Smad-7 overexpression inhibits smad-3 phosphorylation, which decreases TGF-mediated fibrogenesis and protects against liver damage^40^, consequently, smad-7 acts as a negative regulator of HSC activation and hepatic fibrosis. Loss of smad-7 has been reported in fibrotic liver and during HSC activation induced by TGF-*β*1^41^.

Our results not only provide a new insight into the role of miRNA-activated TGF-*β*1/smad and Wnt/β-catenin signalling in liver fibrosis but also show a new anti-fibrotic mechanism of dasatinib in liver fibrosis. The current study demonstrates that dasatinib can down-regulate miR-17 expression, leading to the restoration of WIF1 and smad-7 which further cause the inhibition of both Wnt/β-catenin and TGF-β/smads signalling. In addition, dasatinib can upregulate miR-378a leading to decrease in Wnt-10 expression which contributes to the suppression of activated HSCs. To sum up, we suggest that dasatinib can be a potential therapeutic drug for liver fibrosis due to its crucial role in suppressing various fibrotic signalling pathways and its ability to suppress HCS activation and EMC deposition.
